# Spontaneous tumor regression mediated by human T cells in a humanized immune system mouse model

**DOI:** 10.1038/s42003-023-04824-z

**Published:** 2023-04-22

**Authors:** A. K. Patel, Ankur Dhanik, Wei Keat Lim, Christina Adler, Min Ni, Yi Wei, Maggie Zhong, Cindy Nguyen, Jun Zhong, Yi-Fen Lu, Gavin Thurston, Lynn Macdonald, Andrew Murphy, Cagan Gurer, Davor Frleta

**Affiliations:** 1 Regeneron Pharmaceuticals, Inc., 795 Old Saw Mill River River Road Tarrytown, Tarrytown, NY 10591 USA; 2 Gritstone Bio, 40 Erie St., Cambridge, MA 02139 USA; 3 Eli Lilly and Company, 450 E 29th St., New York, NY 10016 USA; 4 TScan Therapuetics, 830 Winter St., Waltham, MA 02451 USA

**Keywords:** Lymphocytes, Tumour immunology, Animal biotechnology

## Abstract

Immunodeficient mice reconstituted with a human immune system (HIS mice) give rise to human T cells, which make them an attractive system to study human immune responses to tumors. However, such HIS mice typically exhibit sub-optimal responses to immune challenges as well as fail to develop antigen-specific B or T cell memory. Here we report HIS mice mediate spontaneous regression of human B cell lymphoma Raji. Tumor regression was dependent on CD4+ and CD8+ T cell responses and resulted in T cell memory. The T cell memory elicited was mainly Raji-specific, however some level of cross-protection was also elicited to a related B cell lymphoma cell line Ramos. Single-cell RNAseq analysis indicated activation of CD8+ T cells in regressing Raji tumors as well as clonal expansion of specific T cell receptors (TCRs). Cloning of TCRs from Raji-infiltrating T cells into a Jurkat reporter cell line showed reactivity specific for Raji tumor cells. Overall, we report a platform for studying in vivo human T cell tumor immunity by highlighting spontaneous Raji tumor regression, clonal TCR expansion, and T cell memory in HIS mice.

## Abstract (web summary)

Raji tumor spontaneously regresses via human T cell-mediated tumor control in human immune system-reconstituted mice.

## Introduction

Mice with a human immune system (HIS mice) have proven to be an invaluable tool for both modeling human tumor biology and for pre-clinical testing of tumor-eradicating therapeutics. Specifically, immunodeficient mice can be reconstituted with isolated human CD34+ hematopoietic stem cells (HSCs) and give rise to human B cells, T cells, macrophages, NK cells, and granulocytes^1–4^. Recent advances that humanized critical cytokine genes for hematopoietic development have enhanced levels and function of specific innate immune cells. For example, humanization of M-CSF, GM-CSF, and IL-3 have improved levels of human myeloid cells in HIS mice that are valuable in studying the effects of tumor-associated macrophages^2,3^. Humanization of IL-15 increases human NK cells in HIS mice allowing for a better model to test antibody-dependent cellular cytotoxicity (ADCC) of implanted tumors^1^.

Whereas there have been promising advances in human innate immune cells in HIS mice, the functionality of adaptive immune cells such as B and T cells remains a challenge. Even though HIS mice can be successfully reconstituted with physiological levels of B and T cells, antigen (Ag)-specific responses by B and T cells are sub-optimal^5–10^. Though HIS mice have been successfully used to study Ag-specific T-cell responses against pathogens such as HIV and EBV^11–13^, tumor-specific responses have not been observed. Allogenic tumor lines can be implanted and grow in HSC-engrafted HIS mice, highlighting the lack of functional T-cell responses against such allogeneic tumor lines. Indeed, human T cells from HIS mice fail to reject implantation of allogeneic embryonic stem (ES) cells^8^. Overall, even though there is an advantage of such sub-par T-cell responses (engraftment of allogeneic tumor lines or patient-derived tumor sample in HIS mice, study of ES cell interaction, etc.), these results emphasize the dysfunctionality of human T cells in HIS models and point to a need to improve T-cell responses in order to develop a more credible model of the human immune system.

Human T cells in HIS mice also exhibit an abnormal phenotype by expressing higher levels of checkpoint molecules such as PD-1 relative to healthy human T cells, suggesting that the T cells are “over-activated” or “exhausted”^14,15^. This observation has previously been highlighted to indicate how T cells in HIS mice exhibit a phenotype that is reflective of T cells in cancer patients and thus may be an opportunity to test novel cancer immunotherapies^15,16^. Another theory behind human T-cell dysfunction in HIS mice is the idea that they are predominantly selected on murine MHC class I and II. HIS model mice that are also implanted with human bone marrow, fetal liver and fetal thymus (BLT model) can mount anti-viral T-cell responses when infected with HIV-1^17–20^. This BLT model of HIS mice allows for better human T-cell selection being to some degree mediated by the human thymus, such that human HLA selection is enhanced^17–20^. However, there is no indication of HIV-specific memory being elcited^17–20^. In addition, humanization of the mouse MHC to human HLA has been reported to overcome some challenges in T-cell development, but nonetheless antigen-specific T-cell memory remains undetermined and there is relatively poor T-cell help for B-cell humoral immunity^8,21–23^.

Despite challenges with human T-cell biology in HIS mice, a significant advantage of these T-cell limitations is the opportunity to study the limitations of T-cell-specific tumor control within such mouse models^15,16^. One of the challenges in generating tumor-specific T-cell responses is that T cells from cancer patients frequently exhibit similar lack of stimulatory potential as that observed in HIS mice^16^. Along those lines, high PD-1 expression and sub-optimal anti-tumor responses have been described in T cells from cancer patients, and these correspond to greater tumor morbidity^16^. New immune-therapies are aimed at stimulating anti-tumor T-cell responses either through blockade of checkpoint inhibitors such as PD-1 and CTLA-4 or through the use of T cells with chimeric Ag receptors (CAR-T) that can respond to native Ag on tumor cells without MHC-mediated Ag presentation^24–27^. Because of the dysfunctional phenotype observed in human tumor T cell such as higher checkpoint inhibitor expression, HIS mice are a good model for pre-clinical testing of such immune-therapies^16^.

A key remaining consideration is whether human T cells in HIS mice can mount tumor-specific responses at all, which would underline the potential for enhancing these responses with immune-therapies. Since previous work has indicated that even allo-specific T-cell responses are sub-optimal in HIS mice^8^, T-cell responses against allogenic tumors remains to be determined. To that end, we demonstrate that human T cells in our HIS mouse models (herein referred to as SRG: human SIRPα/Rag2−/−/IL-2Rγc−/− or SRG-15: human SIRPα/Rag2−/−/IL-2Rγc−/− human IL-15) can mount a natural tumor-specific response against Raji tumor cells (an allogeneic B-cell lymphoma) that results in rejection of growing Raji tumors in SRG or SRG-15 mice. We also demonstrate that T-cell memory against Raji tumor is elicited, which is one of the first indications of cell-specific T-cell recall response in HIS mice. Furthermore, we characterized this effect functionally through Raji-specific ELIspot analysis and phenotypically through both FACS analysis and single-cell RNAseq of the Raji-specific T cells. In addition, we demonstrate that we can identify Raji-specific TCR repertoires from tumor-infiltrating T cells (TILs). Such advances highlight the use of these models to clone specific TCRs and characterize T cells in HIS mice that can guide future immune-therapies against cancers.

## Results

### HIS mice show strong growth control and memory response against the Raji human tumor cell line

**Fig. 1:**
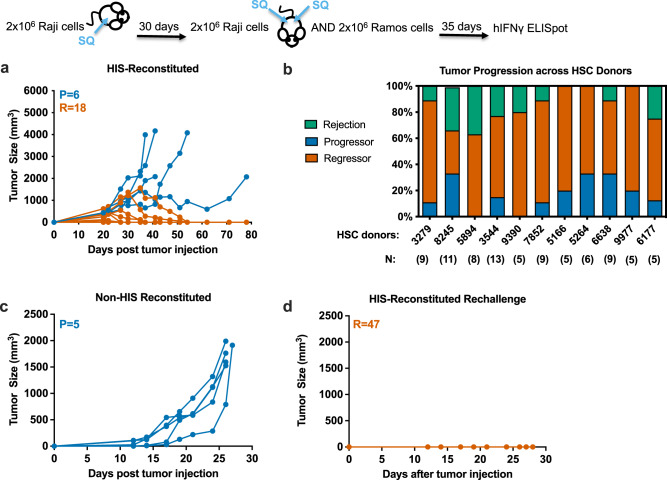
HIS mice show strong growth control and memory response against the Raji human tumor cell line. **a** Newborn pups were given a sublethal irradiation dose 4–24 h prior to an intrahepatic injection of human fetal liver (FL) derived CD34+ cells. Human immune system (HIS) reconstitution was confirmed 10–12 weeks post-engraftment by checking human CD45 levels by flow cytometry. Mice were implanted subcutaneously into the right flank with Raji tumor cells, and tumor growth outcome was recorded (*n* = 24). Progressor (P; blue) is defined as tumor growth only. Regressor (R; orange) is defined as having two tumor volume values smaller than the preceding peak value. Rejection is defined as having two tumor volume values where the second value is zero. Regressor group: R; combined regressor and rejection groups: R. **b** The 80 mice were reconstituted with hematopoietic stem cells from 11 human donors. **c** Non-HIS-reconstituted mice were implanted with Raji tumor (*n* = 5). **d** Mice that rejected Raji tumor during the primary challenge were rechallenged with Raji cells (*n* = 47).

Tumor outcome is defined as Progressor (P), Regressor (R), or Rejection (R). Progressor is tumor growth only. Regressor is defined as having two tumor volume values smaller than the preceding peak value. Rejection is defined as having tumor volume values indicating that the tumor has receded completely. Tumor outcomes of regressor and rejection are combined as R. HIS-reconstituted mice that are implanted subcutaneously with Raji tumor begin to develop tumor around 12 days post-implantation. Tumor regression can be observed starting two weeks after implantation (Fig. 1a). Figure 1a shows a sample experiment in which Raji tumor regression or rejection predominantly occurs, and this result is observed with at least 10 different HSC donors used for HIS reconsititution (Fig. 1b). HIS reconstitution is necessary for spontaneous Raji tumor regression. When non-HIS mice are implanted with Raji tumors, tumor progression is seen in all mice (Fig. 1c). HIS reconstitution allows for spontaneous Raji-specific tumor regression in a majority of HIS-reconstituted mice (Fig. 1b). This effect is in contrast to another allogeneic lymphoma that do not elicit spontaneous regression in HIS mice (Supplementary Fig. 1). HIS-reconstituted mice that have previously cleared Raji tumors over 1 month prior were rechallenged with Raji tumors and no tumor development was observed (Fig. 1d), suggestive of tumor-specific memory. It should be noted that these results were observed with >10 different HSC donors used for HIS reconsititution, which indicates a HIS T-cell response that is allogeneic because most HSC donors have mismatched HLA alleles relative to Raji cells (Supplementary Fig. 2).

### Raji tumor control is T-cell-mediated

**Fig. 2:**
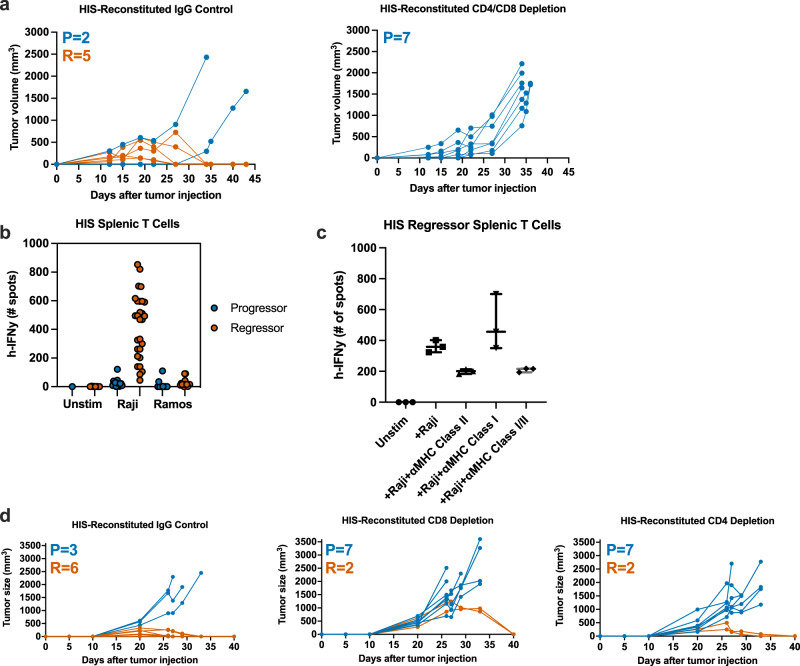
Raji tumor control is T-cell-mediated. **a** Tumor growth curve of mice in the control group (*n* = 7 isotype) compared to CD8-depleted and CD4-depleted (*n* = 7 anti-CD4/anti-CD8). **b** ELISpot data comparing human immune system (HIS) splenic T cells from progressor and regressor mice (*n* = 67). HIS splenic T cells were co-cultured alone (unstimulated), with Raji or with Ramos. **c** ELISpot data of HIS splenic T cells isolated from mouse with Raji regression. T cells were co-cultured alone (unstimulated) or with Raji alone, Raj i+ anti-MHC class II blocking, Raji + anti-MHC class I blocking antibody, and Raji + both anti-MHC class 1/class 2 blocking antibodies. **d** Tumor growth curve of mice in the control group (*n* = 7 isotype), versus CD8-depleted (*n* = 7 anti-CD8) and compared to CD4-depleted (*n* = 7 anti-CD4). The number of mice with tumor progression (P; orange) or regressor (R; blue) is shown. Antibody treatments started 2 days before tumor implantation. Mean value with SD are indicated. Error bars are equivalent throughout.

To investigate if the response was Raji-specific T-cell memory, T-cell depleting antibodies were administered in Raji-challenged HIS mice alongside control mice. Mice with depleted T cells showed tumor progression while the control mice showed predominantly tumor regression, consistent with previously collected data (Fig. 2a). The Raji tumor progression in all mice without CD4/CD8 cells indicates T cells are necessary for Raji tumor control in HIS mice. In accordance with Raji tumor activating tumor-specific T-cell responses, isolated splenic T cells from Raji-challenged mice with regressing tumors exhibited Raji-specific reactivity in an IFNγ ELISpot assay (Fig. 2b). The T-cell reactivity was specific for Raji because Ramos cells, another human B-cell lymphoma, did not elicit an IFNγ response (Fig. 2b). Furthermore, less IFNγ responsiveness was observed with T cells from mice that elicited Raji tumor progression (Fig. 2b). We evaluated whether Raji-induced IFNγ production is dependent on MHC class or MHC class II recognition of Raji cells by isolated T cells. The Raji-stimulated IFNγ response was significantly reduced by MHC class II blocking antibody but not by MHC class I blocking antibody (Fig. 2c), indicating that in vitro IFNγ production in response to Raji is primarily mediated by CD4+ T cells from mice with Raji regression. To determine which T-cell subset is necessary for the control of Raji tumors, CD4+ or CD8+ T cells were depleted in separate cohorts of mice and outgrowth was elicited in HIS-reconstituted mice depleted of either CD4+ or CD8+ T cells (Fig. 2d). The need for both CD4+ and CD8+ T cells to control Raji tumors highlights the importance of CD4+ T cells for CD8+ T-cell memory^28–30^^.^

### Tumor-specific T-cell memory elicited by Raji implantation

**Fig. 3:**
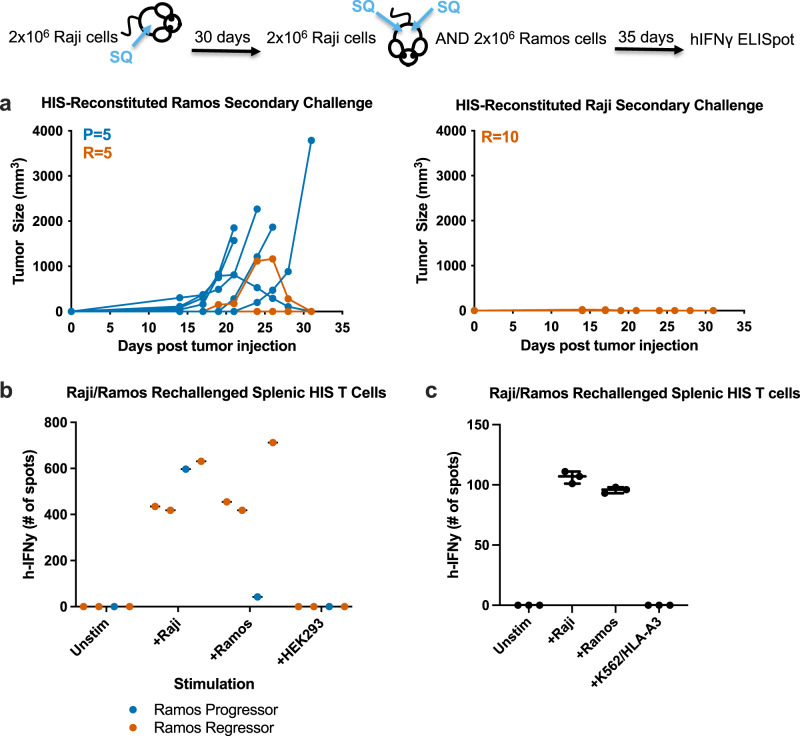
Tumor-specific T-cell memory elicited by Raji implantation. **a** Human immune system (HIS) reconstituted mice were implanted subcutaneously into the right flank with Raji tumor cells. 30 days after tumor clearance, mice (*n* = 10) were rechallenged with Raji in the right flank and Ramos in the left flank. The number of mice with tumor progression (P; blue) or regressor (R; orange) is shown. **b** ELISpot data comparing HIS splenic T cells from Ramos progressor (*n* = 1) and Ramos regressor (*n* = 3) mice. HIS splenic T cells were co-cultured alone (unstimulated), Raji, Ramos or HEK293. **c** ELISpot data of T cells isolated from Raji-experienced mouse that elicited Ramos regression. T cells were co-cultured alone (unstimulated) or with Raji, Ramos, or K562 cells expressing HLA-A3. Mean values with SD are indicated. Error bars are equivalent throughout.

Unlike Raji tumors, Ramos (also a human B-cell lymphoma) implanted HIS mice have tumor progression (Supplementary Fig. 1). All Ramos-challenged mice experienced tumor progression (Supplementary Fig. 1). Raji-experienced mice provided some protection against Ramos tumors (Fig. 3a). Raji-experienced mice dual injected with Ramos and Raji showed Raji rejection in all of the mice (1 mouse had a very small tumor develop before complete rejection) and Ramos tumor control in 50% of the mice (Fig. 3a). Thus, the established Raji-specific T-cell memory provided some tumor control against Ramos. Purified T cells from mice with Raji control and Ramos progression show strong IFNγ response to Raji, but none to Ramos (Fig. 3b). The lack of Ramos response correlates with Ramos progression. Purified splenic T cells from Raji-experienced mouse showing Raji tumor control and Ramos rejection demonstrate IFNγ ELISpot response to both Raji and Ramos tumor (Fig. 3b). Raji and Ramos both share the HLA-A3 MHC allele indicating that T-cell response against Ramos in Raji-experienced mice may be directed against the common alloantigen. However, T cells isolated from Raji-experienced mice that exhibited Ramos regression did not respond via IFNγ ELISpot to K562 cells expressing HLA-A3, even though IFNγ responses were generated to Raji and Ramos tumor cells (Fig. 3c). Thus, Raji T-cell memory may not be directed against HLA-A3 exclusively.

### Characterization of tumor-infiltrating T cells from regressing Raji tumors

**Fig. 4:**
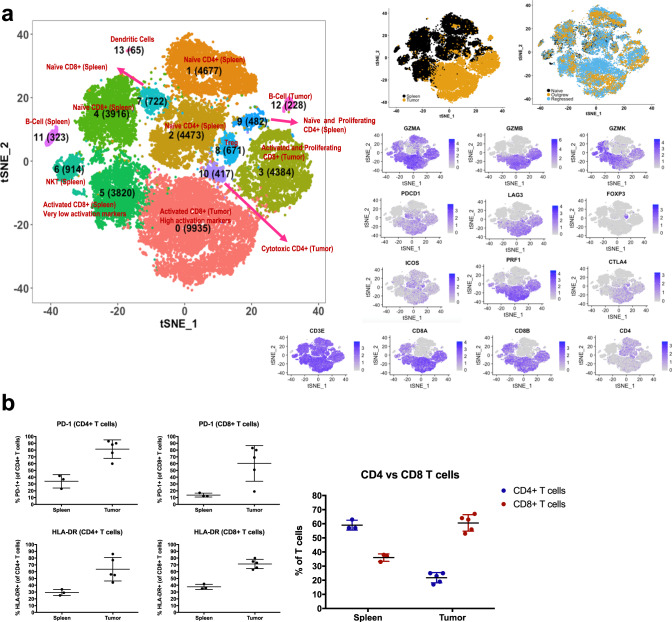
Characterization of tumor-infiltrating T cells from regressing Raji tumors. **a** Human immune system (HIS) reconstituted mice were challenged with Raji (*n* = 13). The spleens and tumors were harvested from regressors and processed for FACS sorting of total hCD45+/hCD3+ T cells. Tumor-derived and splenic T cells were analyzed for VDJ and whole transcriptome using 10X genomics approach. The t-SNE plots show localization of spleen versus tumor, naive versus progression versus regression and highlights specific activation markers. **b** The spleen and matching tumor were harvested from the Raji-challenged HIS-reconstituted mice (*n* = 5) the same day tumor rejection was observed. Both splenic T cells and tumor T cells were stained with antibodies to look at HLA-DR/CD4+ T cells, HLA-DR/CD8+ T cells, and PD-1/CD4+ T cells and PD-1/CD8+ T cells. Mean value with SD are indicated. Error bars are equivalent throughout.

To more comprehensively analyze T cells involved in mediating Raji tumor rejection, we purified T cells from Raji tumors (both regressing and progressing tumors) as well as T cells from matching spleens for single-cell RNA-seq analysis with concurrent TCR analysis. The t-SNE plotting of transcriptional data indicated that tumor-infiltrating T cells in rejecting Raji tumor were predominantly CD8+ T cells expressing activation and effector molecules such as granzyme A (GZMA) whereas matching splenic T cells were predominantly naive CD4+ T cells (Fig. 4a). Interestingly, there was a small population of Foxp3+ Tregs observed in the tumors however, these did not appear to coincide with progressing tumors (Fig. 4a). Furthermore, a small population of cytotoxic CD4+ T cells is observed in rejecting tumors and it remains to be determined to what degree this population controls tumor progression (Fig. 4a). FACS analysis of tumor-infiltrating T cells confirmed data from single-cell RNA-seq analysis in that CD8+ T-cell frequency in rejecting tumors was greater that in matching spleens (Fig. 4b). In addition, both CD4+ and CD8+ T cells in rejecting tumors had higher HLA-DR and PD-1 expression than in concomitant spleens (Fig. 4b), indicating greater T-cell activation in the tumor infiltrate. Overall, this suggests that Raji tumor control is mediated greatly by activated CD8+ T cells.

### TCR frequency and clonality

**Fig. 5:**
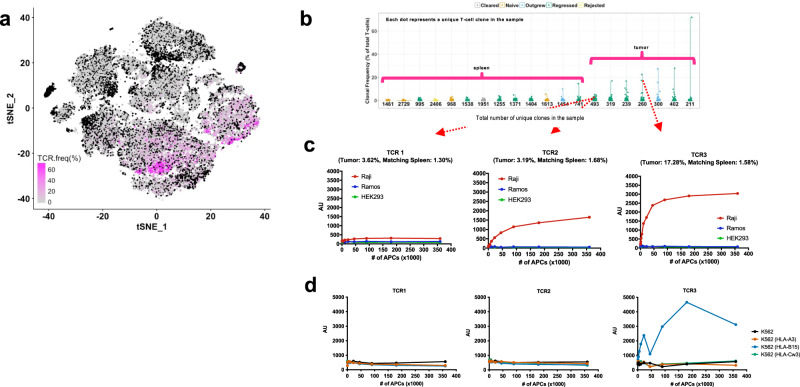
TCR frequency and clonality. **a** The splenic T cells and tumor-infiltrating lymphocytes (TILs) were sorted. Raji-specific TCRs in expanded T cells were identified using TCR repertoire sequencing data. The t-SNE plot depicts TCR frequency in splenic and tumor T cells from 13 regressors. **b** The number of unique clones from the 13 regressors is depicted by a violin plot. **c** Four clonotypes from two regressors tumors were cloned into a Jurkat-luciferase reporter line. The activity against Raji, Ramos and HEK293 is shown from two of the TCR clones. **d** Luciferase assay was used to explore the reactivity of the TCR clones to Raji HLA’s: A3, B15, and CW3.

TCR analysis indicated TCR clonal expansion in rejecting tumors relative to concomitant spleens (Fig. 5a, b). Three TCRs that were greatly expanded in regressing Raji tumors were successfully cloned into Jurkat reporter cells (Fig. 5b). All 3 TCRs showed Raji-specific reactivity and did not respond to Ramos (Fig. 5c). Since it is known that Raji-experienced mice can to some degree control subsequent challenge with Ramos (Fig. 3b, c), it is possible that certain expanded TCR clonotypes in rejecting Raji tumors are also Ramos-reactive, but this experiment only cloned three TCR clonotypes that are expanded in rejecting Raji tumors. The three TCR clonotypes were tested against K562 cells that express HLA-A3, HLA-B15, and HLA-C3, which correspond to the MHC class I molecules expressed by Raji cells. Of the three TCR clonotypes, one was found to be allo-specific against HLA-B15 (Fig. 5d).

Since Raji tumors can elicit a strong T-cell response in vivo, we also tested if Raji cells could stimulate HIS T cells from non-tumor-challenged mice in vitro. This experiment was performed in combination with anti-CD3/CD28 agonist antibodies, strong T-cell stimuli. As discussed previously, HIS T cells are dysfunctional relative to normal healthy human PBMC-derived T cells. Specifically, a strong IFNγ ELISpot response is elicited from human PBMC-derived T cells with anti-CD3/CD28, but isolated HIS T cells do not respond to anti-CD3/CD28 stimulation (Supplementary Fig. 3). In contrast, HIS T cells incubated with Raji cells and anti-CD3/CD28 exhibited a strong IFNγ ELISpot response, and this effect was specific for Raji cells since Ramos cells, A20 cells, (a murine lymphoma), and HEK293 cells, (human epithelial cells), did not recapitulate this “helper” function of Raji cells towards T-cell activation (Supplementary Fig. 3). Control anti-CD3/CD28 stimulation of Raji cells alone indicated that IFNγ was being made by T cells (Supplementary Fig. 3).

Because of Raji cells’ unique ability to render HIS T cells responsive to classical T-cell stimuli in vitro and indeed elicit tumor-specific memory in vivo, we explored what factors on Raji provided this HIS T-cell stimulatory effect. Like many B-cell lymphomas, Raji cells express a wide assortment of T-cell costimulatory molecules such as CD80 and CD86. We took advantage of the differences between Raji and Ramos cells in eliciting HIS T-cell activation and observed that Raji cells expresses much greater higher levels of the TNF superfamily member CD70 than Ramos (Supplementary Fig. 4A). CD70 has previously been reported to activate CD4+ and CD8+ T cells and contribute to CD8+ T-cell memory^31–33^. Blocking CD70 with antagonistic antibodies greatly reduces Raji cell stimulation of non-tumor challenged HIS T cells treated with anti-CD3/CD28 in vitro (Supplementary Fig. 4b). This effect is observed with HIS T cells from two different HSC donors (Supplementary Fig. 4b). Furthermore, Raji cells with CD70 deleted also have less stimulatory capacity in vitro than control Raji cells expressing CD70 (Supplementary Fig. 4c).

The in vitro data would suggest that the CD70-CD27 axis contributes greatly to T-cell-mediated control of Raji tumor in vivo. However, Raji-CD70 KO tumors were rejected in HIS-reconstituted mice comparably to control Raji tumors (Supplementary Fig. 4d). Indeed, Raji tumors with CD80/86/70 deletion also rejected in HIS-reconstituted mice (Supplementary Fig. 4d), indicating that other factors may contribute to the ability of Raji tumors to elicit tumor-specific T-cell responses in vivo.

## Discussion

We have demonstrated that Raji tumors can grow and eventually regress in our HIS mice mouse models and that this tumor control is mediated by Raji-specific human T-cell responses. In this regard, the HIS mouse models used were Rag2−/− IL-2R_γ_−/− mice with humanized SIRPα (SRG) or with additional humanization of IL-15 (SRG-15) which increases human NK cell development^4^. Since tumor control is primarily mediated by T cells, Raji regression is comparable between SRG and SRG-15 mice, despite the latter strain having more NK cells. Thus, we refer to all data has being observed in HIS mice because the findings are identical between SRG and SRG-15 strains. Although, we recognize that more extensive profiling of T-cell responses, especially Raji-specific TCRs, may be warranted between the two strains.

Importantly, T-cell memory is established against Raji tumors that can control subsequent re-challenge in SRG or SRG-15 mice that have cleared Raji tumors during primary challenge. To the best of our knowledge, this is the first time that a functional human T-cell memory response to specific tumors has been established in HSC-engrafted HIS mice^10,14–16^. These results are observed with at least ten different HSC donors for HIS reconstitution with both Raji and Ramos cells, which are allogeneic tumors relative to the human T cells in these mice. However, only Raji cells can elicit spontaneous regression from the HIS T cells, indicating that Raji can unqiuely activate this allo-specific response.

During secondary re-challenge of SRG or SRG-15 mice that have cleared Raji tumors, there is some level of cross-protection against Ramos tumor indicating Raji-specific T-cell memory that is established can also be reactive against Ramos tumor. This result is further emphasized by ELISpot data that showed that when mice that cleared Raji tumor were rechallenged with Ramos, mice that went on to clear Ramos tumor had Ramos-specific IFNγ+ T cells, whereas mice that exhibited Ramos progression did not show Ramos-specific T-cell responses. It should be noted that the IFNγ+ T cells in these responses are probably CD4+ T cells, due to the IFNγ ELISpot response being blocked by anti-MHC class II blocking antibody. As our depletion studies have shown, both CD4+ and CD8+ T cells are necessary for tumor control. We surmise that elimination of tumor is mediated by CD8+ cytotoxic CD8+ T cells because (1) the greater percentage of CD8+ T cells infiltrating the regressing Raji tumor and (2) the activation profile of the CD8+ T cells in our single-cell RNA-seq analysis that is indicative of cytotoxic potential. In addition, we have cloned several Raji-specific TCRs from TILs in these mice through single-cell analysis. However, none of the TCRs were Ramos-reactive, despite Raji and Ramos sharing the HLA-A3 allele. The recognition by these clones is dependent on CD8-MHC class I since the reporter line expreses human CD8. We focused on the most highly expanded TCRs and it may be worthwhile to examine other TCR sequences from TILs in Raji-implanted SRG-15 mice to identify ones that exhibit cross-reactivity to other tumors.

One of the advances of the SRG-15 HIS mouse model is increased development of human NK cells that are reflective of normal human levels, as opposed to the sub-optimal levels observed in SRG mice, in which human NK cells are ~10% of the levels seen in normal human PBMCs (4). The humanization of IL-15 improves the development of human NK cells (4). Though human NK cells are increased in this HIS model, we do not believe that they are the primary mediators of tumor control. First, Raji tumor regression kinetics are similar between SRG and SRG-15 HIS strains; the latter has 10x higher numbers of human NK cells in the spleen and blood. Second, selective depletion of human T cells in SRG or SRG-15 mice abolishes Raji-specific tumor regression and results in progression. This finding does not of course preclude the possibility that Raji-specific T cells are stimulating the NK-mediated killing of Raji through cytokines, receptor engagement, etc. Lastly, around 90% of human CD45+ cells that infiltrate Raji tumors in SRG-15 mice are T cells. Once again, all of these data do not preclude an indirect effect of NK cells in controlling Raji tumors. SRG-15 mice were previously used to study ADCC against implanted Raji tumor by human NK cells (4). It should be noted that this study showed the increased efficacy of ADCC against Raji in SRG-15 due to increased NK cells (4); however, the killing of Raji tumor by ADCC was fairly quick so tumor was only measured as far as 16 days, whereas Raji regression driven by human T cells generally begins to occur past 20 days post tumor implantation. Thus, longer tumor growth studies are needed to observe T-cell-mediated control of Raji. However, these data also highlight how this model can be used to evaluate immune therapeutics to accelerate tumor-specific T-cell responses, especially if such responses can lead to T-cell memory.

Characterization of Raji-infiltrating T cells both by FACS analysis and single-cell RNAseq indicated that human T cells in tumor exhibit more of an activated phenotype relative to T cells from adjoining spleen. Specifically, we have observed higher PD-1 and HLA-DR+ T cells in Raji tumor than human T cells in matching spleen. Comprehensive analysis of infiltrating T cells by single-cell RNAseq revealed the expression of an activated molecular pattern. Furthermore, human T cells from mice that exhibited Raji rejection had higher T-cell activation status and effector phenotype than T cells from mice that demonstrated Raji progression. Molecular profiling of infiltrating human T cells in this tumor model further reveals potential T-cell molecules, such as checkpoint inhibitors, that can be targeted to potentiate tumor-specific T-cell responses, especially since this molecular profiling also elucidates expanded tumor-specific TCRs.

Of the Raji-specific TCRs that were cloned from infiltrating T cells isolated from rejecting tumors, only one TCR was shown to be allo-specific. As mentioned previously, some expanded TCRs should also be reactive to Ramos tumors, but these were not detected in current study. More comprehensive cloning of expanded TCRs are needed to garner a better understanding of antigen specificity of T cells that control Raji tumors in vivo. It may be worthwhile to clone TCRs from memory T cells (CD45RO+) that are elicited after Raji challenge. Nonetheless, these studies highlight how this technology can be used to determine and isolate tumor-specific TCRs, esp. from T cells that overcome the dysfunctionality that is commonly observed in tumor patients.

As previously discussed, human T cells in HSC-engrafted HIS mice are dysfunctional both in phenotype and due to sub-optimal Ag-specific response. The fact that Raji tumor can elicit a potent Raji-specific human T-cell response, induce specific TCR clonal expansion, and instill Raji-specific T-cell memory emphasizes the uniqueness of Raji lymphoma cells in stimulating HIS T cells. Indeed, Raji cells stimulated isolated human T cells in vitro from non-tumor-challenged SRG-15 mice. Whereas using potent anti-CD3/CD28 agonists stimulate healthy human PBMC-derived T cells, comparable stimulation of isolated human T cells from SRG or SRG-15 mice fails to elicit T-cell activation unless performed concurrently with Raji cells. This finding is consistent with a unique ability of Raji lymphoma cells to elicit T-cell-mediated tumor control in vivo. In accordance with this, Ramos cells, a related lymphoma line, does not elicit T-cell-mediated tumor control in SRG or SRG-15 mice and does not stimulate T cells in vitro from non-tumor challenged mice. These results highlight the need to determine what Raji-specific factors drive such potent human T-cell activation in HIS mice. CD70 is a costimulatory molecule that is a member of the TNF superfamily^31–33^. Interaction of CD70 from APCs with CD27 on T cells has been shown to induce CD4+ T-cell differentiation to Th1 or Th17 cells as well as to promote CD8+ T-cell activation and memory differentiation^31–33^. Preliminary in vitro analysis has indicated that blocking CD70 or CD27 can mitigate Raji-specific T-cell activation, however, in vivo data with CD70 KO Raji cells indicated that T-cell-mediated tumor control is not solely dependent on the CD70-CD27 axis. It remains to be determined what additional factors Raji cells use to stimulate human T cells from HIS mice. Considering that the dysfunction of human T cells in HIS mice appears to be similar to the T-cell dysfunctionality observed in cancer patients, such analysis could unveil novel targets to stimulate anti-tumor T-cell responses.

## Methods

### Human CD34+ cell isolation

Human fetal liver (FL) samples were obtained from Advanced Biosciences Resources (Alameda, CA) with proper consent. FL samples were cut in small fragments, treated for 25 min at 37 °C with Collagenase D (100 ng/mL; Roche). The cell suspension was prepared, and the human CD34+ cells were separated by density gradient centrifugation, followed by positive immunomagnetic selection using anti-human CD34 microbeads according to the manufacturer’s instructions (Miltenyi Biotec). Cells were either frozen in 10% DMSO containing human albumin serum and stored in liquid nitrogen or injected directly.

### Mice

Immunodeficient mice with Rag2 and IL-2Rγ deletions were generated and bred in-house. Two lines were used: SRG: Balb/cAnN;129S4/SvJae - Rag2^–/−^ human Sirpα IL-2Rγ^−/−^ and SRG-15: Balb/cAnN;129S4/SvJae - Rag2^−/−^, human Sirpα, IL-2Rγ^−/−^, human IL-15. Mice were maintained on a sulfa diet (LabDiet, St. Louis, MO) in a MPF facility, and were intra-bred for about 10-11 generations. All mice in the study were 5–7 months of age at the start of the experiment. A mix of male and female were used for all experiments. All experiments were done in compliance with Regeneron Institutional Animal Care and Use Committee protocols.

### Human CD34+ immune cell reconstitution

The generation of knock-in mice encoding human SIRPa And IL-15 in a 129xBALC/c genetic background were created by Velocigene technology. Mice were crossed to a Rag2^−/−^ IL2Rg^−/−^ background and will be abbreviated as follow: SRG (=S^h/m^RG) and SRG-15 (= S^h/m^RG-15^h/m^). Newborn pups were sublethally irradiated (360 cGy; X-RAD 320 irradiator) 4–24 h prior to an intrahepatic injection of 1 × 10^5^ human FL-derived CD34+ cells.

### Analysis of human hematopoietic cell populations

Blood was collected retro-orbitally 10–12 weeks after engraftment. Red blood cells were lysed using ACK (Gibco) and the cells were stained with the following monoclonal antibodies from Invitrogen and BD for flow cytometry analysis. For overall hematopoietic engraftment: anti-mouse CD45-APC-Cy7 (30-F11, 1:100), anti-human CD45-PE-Cy5.5 (HI30, 1:100), anti-human CD19-FITC (HIB19, 1:100), anti-human CD3-PB (S4.1, 1:20), anti-human NKp46-APC (9E2, 1:20), anti-human CD14-PE-Cy7 (M5E2, 1:33) and anti-human PD-1-BV605 (EH12, 1:40). The samples were acquired by a Fortessa (BD Biosciences) or Symphony (BD Biosciences) and analyzed using FACSDiva (BD Biosciences) and FlowJo software. Mice with ≥10% hCD45+ of total circulating CD45+ cells (total including both mouse and human CD45+ cells) were used for experiments. For experimental repeats, different donor sources of human CD34+ cells were used. Donor-to-donor variations were comparable with the range of variation between individual same donor CD34+ cell-engrafted mice.

### Cell culture

Raji (CCL-86; ATCC) and Ramos (CRL-1596; ATCC) were grown as monolayer cultures in RPMI 1640 supplemented with 10% heat-inactivated fetal calf serum (Gibco), penicillin (100 units/mL), streptomycin (100ug/mL), and l-glutamine (200 mM) at 37 °C in a humidified, 10% CO_2_ incubator. Raji and Ramos tested negative by PCR for mouse viruses and mycoplasma. Cells were authenticated by STR profiling and were 100% matched to the database.

### Tumorigenesis

HIS-reconstituted and non-HIS-reconstituted SRG-15 mice were randomly assigned to treatment groups. Overall, 2 × 10^5^ tumor cells were implanted subcutaneously under anesthesia in the right flank of the mouse. Volume was determined by calipher measurement and calculated using the following formula: tumor volume (mm^3^) = 0.5 × (length × width^2^). Tumor measurements were collected twice a week and evaluated as progressor (tumor growth only), regressor (two tumor volume values smaller than the preceding peak value) and rejection (two tumor volume values where the second value is zero). Progressor group: P; Combined regressor and rejection groups: R.

### T-cell depletion

Mice were injected intra-peritoneally with 200 μg of anti-CD4 (OKT4; BioXcell) and/or 200 μg anti-CD8 (OKT8; BioXcell) monoclonal antibodies at days −2, −1, 0, +1, +6, and +14 relative to Raji tumor implantation. Control mice were given 200 μg mouse IgG2b and/or mouse IgG2a isotype control at same time as anti-CD4 and/or anti-CD8 treatment. Tumor growth was measured then as described above.

### ELISPOT assay

ELISPOT assay was established to determine the frequency of T cells capable of responding to a specific stimulus by secretion of IFNγ. Each well in a 96-well PDVF ELISPOT plate (Millipore) was coated with hIFNγ capture antibody(BD) at 1:200 of phosphate-buffered saline (PBS), pH 7.2 (Gibco). Following overnight incubation at 4 °C, the plate was blocked with 10% fetal calf serum (Gibco) in PBS for 2 h at 37 °C. Splenic T cells were isolated by immunomagnetic selection using anti-human microbeads according to the manufacturer’s instructions (Easy Sep Human T Cell Isolation Kit; StemCell Technologies). Purified T cells were added 2 × 10^5^ per well in triplicates. Tumor cells were added to selected wells at 4:1 T-cell/tumor cell ratio. In some experiments, tumor cells were pre-blocked with 25 μg/mL of pan-MHC class I (clone W6/32; Biolegend) and/or pan-MHC class II (clone Tu83; Biolegend) blocking antibodies. Plate was incubated overnight at 37 °C in a humidified, 10% CO_2_ incubator. Plate was extensively washed in 0.05% Tween 20–PBS and hIFNγ detection (BD) was added to each well at 1:250 of 10%FBS + PBS for two hours at room temperature. Following an extensive washed with 0.05% Tween 20–PBS the wells were coated with Streptavidin-HRP (MABTECH) at 1 μL per mL of 10% FBS + PBS for 45 min at room temperature. The plate was washed with 0.05% Tween 20–PBS extensively. TMB substrate (MABTECH) was added to each well for 10–20 min, until the color developed. Substrate reaction was stopped by washing wells with water. Plate was dried, and the spots were enumerated by AID plate reader and the data was analyzed by GraphPad Prism.

### Tissue homogenization

Raji tumors were minced, and enzymatically digested for 20 min with intermittent agitation using tumor dissociation kit (Miltenyi, 130-096-730) at 37 °C, treated with RPMI and filtered (70-mm nylon filter; Falcon). Spleen was harvested and homogenized. Single-cell suspensions of tumor and spleen were subjected to ACK lysing buffer (Gibco). Tumor and spleen single-cell suspensions were incubated with anti-mouse CD45-APC-Cy7 (30-F11, 1:100), anti-human CD45-PE-Cy5.5 (HI30, 1:100), anti-human CD19-FITC(HIB19, 1:100), and anti-human CD3-PB (S4.1, 1:20). The cells were sorted on Astrios (Beckman Coulter) for human T cells. Sorted cells were used for single-cell sequencing.

### Single-cell preparation and sequencing

Single cells suspended in PBS with 0.04% BSA (~6000 cells) were loaded on a Chromium Single Cell Instrument (10X Genomics). RNA-seq and V(D)J libraries were prepared using Chromium Single Cell v1.0 5’ Library, Gel Beads & Multiplex Kit (10X Genomics). After amplification, cDNA was split into RNA-seq and V(D)J library aliquots. To enrich the V(D)J library aliquot for TCR a/b, the cDNA was split into two 20 ng aliquots and amplified in two rounds using primers designed in-house. Specifically, for first round amplification the primers used were MP147 (ACACTCTTTCCCTACACGACGC) for short R1, MP122 (GGTGCTGTCCTGAGACCGAG) for mouse TRAC, and MP123 (CAATCTCTGCTTTTGATGGCTCAAAC) for mouse TRBC. For second round amplification, 20 ng aliquots from the first round were amplified using MP147 (ACACTCTTTCCCTACACGACGC) for short R1, MP130 (GTGACTGGAGTTCAGACGTGTGCTCTTCCGATCTTGGTACACAGCAGGTTCTGG) a nested R2 plus mouse TRAC, and MP131 (GTGACTGGAGTTCAGACGTGTGCTCTTCCGATCTGACCTTGGGTGGAGTCACATTTCTC) a nested R2 plus mouse TRABC. In the case of human TRAC and TRBC, the procedure was the same as above, but used MP120 (GCAGACAGACTTGTCACTGGA) and MP121 (CTCTGCTTCTGATGGCTCAAACA) for round 1 enrichment and MP128 (GTGACTGGAGTTCAGACGTGTGCTCTTCCGATCT GCAGGGTCAGGGTTCTGGATA) and MP129 (GTGACTGGAGTTCAGACGTGTGCTCTTCCGATCT ATG GCT CAA ACA CAG CGA CCT) for round 2 enrichment. V(D)J libraries were prepared from 25 ng each mTRAC or hTRAC and mTRBC or hTRBC amplified cDNA. Paired-end sequencing was performed on Illumina NextSeq500 for RNA-seq libraries (Read 1 26-bp for unique molecular identifier (UMI) and cell barcode, 8-bp i7 sample index, 0-bp i5, and Read 2 55-bp transcript read) and V(D)J libraries (Read 1 150-bp, 8-bp i7 sample index, 0-bp i5, and Read 2 150-bp read). For RNA-seq libraries, Cell Ranger Single-Cell Software Suite (10X Genomics, v2.2.0) was used to perform sample demultiplexing, alignment, filtering, and UMI counting. The mouse mm10 genome assembly and RefSeq gene model for mice were used for the alignment. For V(D)J libraries, Cell Ranger Single-Cell Software Suite (10X Genomics, v2.2.0) was used to perform sample demultiplexing, de novo assembly of read pairs into contigs, align and annotate contigs against all of the germline segment V(D)J reference sequences from IMGT, label and locate CDR3 regions, group clonotypes.

### Singe-cell data analyses

Single-cell RNA-seq data QC Single cells were filtered for downstream analysis by the following criteria: UMI (unique molecular identifier) count within the range between 500 and 6000 and mitochondria percentage greater than 20% of the total UMI count. 271 cells were filtered out. Gene expression (in UMI) is scale normalized to log1p(UMI.Count*scaling.factor/(total UMI count)), where scaling.factor = 10,000 and log1p is log(1 + x). The variable genes were found with the average expression between 0.0125 and 3.0, with a dispersion greater than 0.5.

All experiments were duplicated. We used the dataset from one experiment for discovery and the other for result validation. For principal component analysis (PCA), the analysis was run on normalized and transformed UMI counts on variably expressed genes. These PC outputs were loaded as input to generate t-SNE plots. Fourteen clusters were defined based on the selected clustering resolution. 3D t-SNE plot was drawn using Rtsne package with default parameters. 2D t-SNE plot was generated with Seurat package. Differentially expressed genes were identified for clustering based on Avg. |log(fold change)| > 0.25 (or, avg. fold change > 1.28 or <0.78) and Wilcoxon rank-sum test (adj. *P* value <0.01) to only include genes that are detected in at least 10% of cells in either of the two populations.

### TCR cloning

Tumor-infiltrating lymphocytes and human T cells were isolated from the tumor and matching spleen, respectively. The splenic T cells and TILs were sorted for CD3 + TCR + cells. Raji-specific TCRs in expanded T cells were identified using TCR repertoire sequencing data. TCR expression constructs were generated by synthesizing gBlock gene fragments (IDT) encoding bicistronic cassettes with the full-length TCRA sequence followed by TCRB, separated by a F2A self-cleaving peptide sequence (full insert sequences are provided in Supplementary Table 1). Fragments were cloned into the pLVX-EF1a-IRES-Zeocin lentiviral vector (Takara) via XbaI and NotI restriction sites.

### TCR reporter lines

TCR sequences were introduced by lentiviral transduction into a parental Jurkat reporter line lacking endogenous TCRα/β expression (via Crispr-mediated disruption), and engineered to express human CD8α, CD8β, and an AP1-responsive luciferase reporter (Qiagen # CLS-011L). Lentivirus was generated by co-transfection of TCR, Gag/Pol, and VSV-G envelope expression constructs into HEK-293T cells (ATCC CRL-3216) using Lipofectamine LTX reagent (Life Technologies 15338100). Cell culture supernatants were harvested 48 h post-transfection, and lentivirus was concentrated using the Lenti-X reagent (Takara 631232) according to the manufacturer’s protocol. Transduction of Jurkat cells was done by adding 20 μl concentrated viral supernatant to 2 × 10^5^ cells in 200 μl total media volume in round-bottom 96-well plates, and spinning plates at 2500 rpm for 90 min at RT. Transduced cells were enriched by antibiotic selection. Surface TCR+ CD8+ CD28+ cells were FACS-sorted and expanded under antibiotic selection media. The reactivity of the sorted clones reactivity was measured against a dose titration of Raji, Ramos, HEK293, K562-HLA-A3, K562-HLA-B15 and K562-HLA-CW3 (# of APCs to 50,000 Jurkat reporter cells) by using One-Glo luciferase assay kit (Promega) according to manufacturer’s instructions.

### Statistics and reproducibility

Tumor implantation experiments were repeated in at least ten different HSC donors. A minimum of 5–7 mice were used in each experimental group. ELISpots had triplicate wells per condition and student *t* tests (unpaired) were performed to compare conditions with a *P* value of less than 0.05, indicating statistical significance.

### Reporting summary

Further information on research design is available in the Nature Portfolio Reporting Summary linked to this article.

## Supplementary information

Supplementary Material

Description of Additional Supplementary Files

Supplementary Data

Reporting Summary

## Data Availability

All data generated or analyzed during this study are included in this published article (and its supplementary information files). Single-cell RNA-seq data reported here has been deposited in Gene Expression Omnibus. The accession code is GSE226662. Source data for figures can be found in Supplementary Data. Please reach out to the corresponding author for any material requests.

## References

[1] Herndler-Brandstetter D (2017). Humanized mouse model supports development, function, and tissue residency of human natural killer cells. Proc. Natl Acad. Sci. USA.

[2] Rongvaux A (2014). Development and function of human innate immune cells in a humanized mouse model. Nat. Biotechnol..

[3] Saito Y (2016). Peripheral blood CD34(+) cells efficiently engraft human cytokine knock-in mice. Blood.

[4] Strowig T (2011). Transgenic expression of human signal regulatory protein alpha in Rag2-/-gamma(c)-/- mice improves engraftment of human hematopoietic cells in humanized mice. Proc. Natl Acad. Sci. USA.

[5] André MC (2010). Long-term human CD34+ stem cell-engrafted nonobese diabetic/SCID/IL-2R gamma(null) mice show impaired CD8+ T cell maintenance and a functional arrest of immature NK cells. J. Immunol..

[6] Kooreman NG (2017). Alloimmune responses of humanized mice to human pluripotent stem cell therapeutics. Cell Rep..

[7] Lang J (2017). Replacing mouse BAFF with human BAFF does not improve B-cell maturation in hematopoietic humanized mice. Blood Adv..

[8] Lee JY, Han AR, Lee DR (2019). T lymphocyte development and activation in humanized mouse model. Dev. Reprod..

[9] Watanabe Y (2009). The analysis of the functions of human B and T cells in humanized NOD/shi-scid/gammac(null) (NOG) mice (hu-HSC NOG mice). Int. Immunol..

[10] Yu H (2017). A novel humanized mouse model with significant improvement of class-switched, antigen-specific antibody production. Blood.

[11] Baenziger S (2008). Human T cell development and HIV infection in human hemato-lymphoid system mice. Curr. Top. Microbiol Immunol..

[12] Gorantla S (2010). CD8+ cell depletion accelerates HIV-1 immunopathology in humanized mice. J. Immunol..

[13] Strowig T (2009). Priming of protective T cell responses against virus-induced tumors in mice with human immune system components. J. Exp. Med..

[14] Park N (2020). Preclinical platform for long-term evaluation of immuno-oncology drugs using hCD34+ humanized mouse model. J. Immunother. Cancer.

[15] Wang M (2018). Humanized mice in studying efficacy and mechanisms of PD-1-targeted cancer immunotherapy. FASEB J..

[16] Capasso A (2019). Characterization of immune responses to anti-PD-1 mono and combination immunotherapy in hematopoietic humanized mice implanted with tumor xenografts. J. Immunother. Cancer.

[17] Brainard DM (2009). Induction of robust cellular and humoral virus-specific adaptive immune responses in human immunodeficiency virus-infected humanized BLT mice. J. Virol..

[18] Claiborne DT (2019). Immunization of BLT humanized mice redirects T cell responses to gag and reduces acute HIV-1 viremia. J. Virol..

[19] Dudek TE, Allen TM (2013). HIV-specific CD8^+^ T-cell immunity in humanized bone marrow-liver-thymus mice. J. Infect. Dis..

[20] Dudek TE (2012). Rapid evolution of HIV-1 to functional CD8^+^ T cell responses in humanized BLT mice. Sci. Transl. Med..

[21] Billerbeck E (2013). Characterization of human antiviral adaptive immune responses during hepatotropic virus infection in HLA-transgenic human immune system mice. J. Immunol..

[22] Danner R (2011). Expression of HLA class II molecules in humanized NOD.Rag1KO.IL2RgcKO mice is critical for development and function of human T and B cells. PLoS ONE.

[23] Masse-Ranson G (2019). Accelerated thymopoiesis and improved T-cell responses in HLA-A2/-DR2 transgenic BRGS-based human immune system mice. Eur. J. Immunol..

[24] Brendel C, Rio P, Verhoeyen E (2020). Humanized mice are precious tools for evaluation of hematopoietic gene therapies and preclinical modeling to move towards a clinical trial. Biochem. Pharm..

[25] Frank AM (2020). Combining T-cell-specific activation and in vivo gene delivery through CD3-targeted lentiviral vectors. Blood Adv..

[26] Johanna I (2019). Evaluating in vivo efficacy—toxicity profile of TEG001 in humanized mice xenografts against primary human AML disease and healthy hematopoietic cells. J. Immunother. Cancer.

[27] June CH (2018). CAR T cell immunotherapy for human cancer. Science.

[28] Borrow P (1996). CD40L-deficient mice show deficits in antiviral immunity and have an impaired memory CD8+ CTL response. J. Exp. Med..

[29] Borrow P (1998). CD40 ligand-mediated interactions are involved in the generation of memory CD8(+) cytotoxic T lymphocytes (CTL) but are not required for the maintenance of CTL memory following virus infection. J. Virol..

[30] Laidlaw BJ, Craft JE, Kaech SM (2016). The multifaceted role of CD4(+) T cells in CD8(+) T cell memory. Nat. Rev. Immunol..

[31] Hendriks J (2000). CD27 is required for generation and long-term maintenance of T cell immunity. Nat. Immunol..

[32] Hendriks J, Xiao Y, Borst J (2003). CD27 promotes survival of activated T cells and complements CD28 in generation and establishment of the effector T cell pool. J. Exp. Med..

[33] Rowley TF, Al-Shamkhani A (2004). Stimulation by soluble CD70 promotes strong primary and secondary CD8+ cytotoxic T cell responses in vivo. J. Immunol..

