# Supplementary material for: Spontaneous tumor regression mediated by human T cells in a humanized immune system mouse model
Source: Commun Biol. 2023 Apr 22;6:444. doi: 10.1038/s42003-023-04824-z (PMC10122651; doi:10.1038/s42003-023-04824-z)
Supplement: Supplementary file 1 — Supplementary Material [file 42003_2023_4824_MOESM1_ESM.pdf]

Suppl Fig 1: Tumor kinetics in B-cell lymphoma lines.

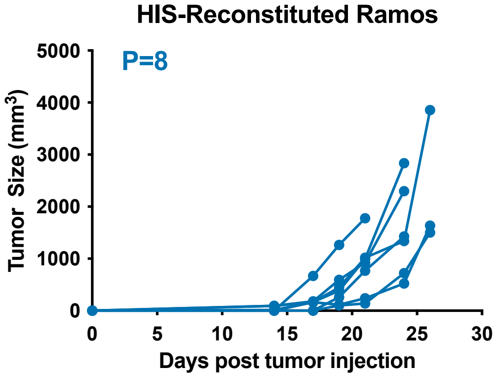

Mice were implanted subcutaneously into the right flank with Ramos tumor cells (n=8). Progressor (P;blue) is defined as tumor growth only.

Suppl Fig 2: Genotype of HSC donors and cell lines.

Donor	A Locus		B Locus		C Locus		DPA1 Locus		DPB1 Locus		DQA1		DQB1 Locus		DRA1		DRB1 Locus	
3279	*02:01:01:01	*23:01:01	*07:02:01	*44:02:01:01	*05:01:01:01	*07:02:01:01	-	-	*01:01:01	*04:01:01:01	-	-	*05:02:01	*06:02:01	-	-	*15:01:01:01	-
5894	01:01	68:02	15:10	08:01	07:01	03:04	-	-	-	-	02:01	02:01	-	-	01:01	01:02	-	-
9390	01:01	03:01	07:02	57:01	07:02	06:02	-	-	-	-	02:01	03:02	-	-	01:01	01:01	-	-
5166	*02:01:01	*29:02:01	*53:01:01	*44:03:01	*04:01:01	*16:01:01	*01:03:01	*01:03:01	*04:01:01	*104:01:01	*01:05:01	*02:01:01	*02:02	*05 01	*01 01	*01 02	*07 01	*12 01
5264	*01 01	*03 01	*14 02	*37 01	*06 02	*08 02	*01 03	*01 03	*04 01	*04 01	*01 02	*05 01	*02 01	*06 09	*01 02	*01 02	*03 01	*13 02
6638	*02:1:	*29:2:	*08:1:1	*45:1:1	*07:1:1	*16:1:1	*01:3:1	*01:3:1	*04:1:1	*04:2:1	*01:2:1	*05:1:1	*02:1:1	*06:2:1	*01:2:2	*01:2:3	*03:1:1	*03:2:3
6177	*02:1:1	*23:17:	*42:1:1	*44:3:1	*16:1:1	*16:1:1	*02:1:1	*02:1:1	105:1:1	*11:1:1	*02:1:1	*05:5:1	*02:2:1	*03:24:	*01:1:1	*01:1:1	*11:2:1	*11:2:1
8245	NA	NA	NA	NA	NA	NA	NA	NA	NA	NA	NA	NA	NA	NA	NA	NA	NA	NA
5894	NA	NA	NA	NA	NA	NA	NA	NA	NA	NA	NA	NA	NA	NA	NA	NA	NA	NA
3544	NA	NA	NA	NA	NA	NA	NA	NA	NA	NA	NA	NA	NA	NA	NA	NA	NA	NA
7852	NA	NA	NA	NA	NA	NA	NA	NA	NA	NA	NA	NA	NA	NA	NA	NA	NA	NA
9977	NA	NA	NA	NA	NA	NA	NA	NA	NA	NA	NA	NA	NA	NA	NA	NA	NA	NA

Cell Line	A Locus		B Locus		C Locus		DPB1 Locus		DQA1		DQB1 Locus		DRB1 Locus	
Raji	*03:01:01		*15:10:01		*03:04:02	*04:01:01	*01:01:01		*01:05:01	*5:01:01	*02:01:01	*05:01:01	*03:01:01	*10:01:01
Ramos	*03:01	*03:01	*44:03	*51:01	*16:01	*16:01	*04:01:01	*104:01:01	*02:01	*02:01	*02:01	*02:01	*07:01	*14:103

Genotype data not available for donors: 8245, 5894, 3544, 7852, and 9977.

Suppl Fig 3: Human Immune System T cells perform sub-optimally in comparison to Human PBMC-derived T cells *in vitro*.

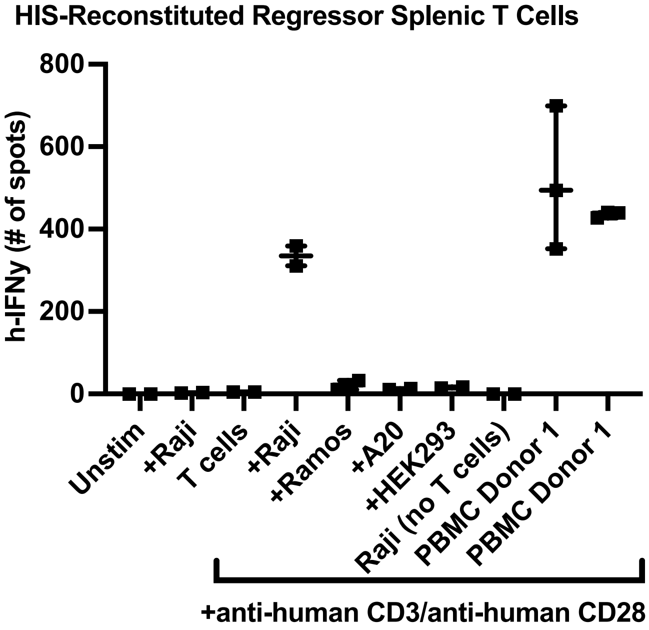

ELISPOT assay was used to determine the frequency of T cells capable of responding to a specific stimulus by secretion of human interfereon-gamma (IFN-γ). Splenic T cells from HIS mice were co-cultured with or without soluble anti-human CD3(OKT3) and anti-human CD28(CD28.2) and tumor cells in an ELISPOT assay. Raji(CCL-86) and Ramos(CRL-159) are human Burkitt's lymphoma. A20(TIB-208) a mouse B lymphocyte was used as a mouse control and HEK293(CRL-1573) a human embryonic kidney was used as nonrelevant human control. Human immune system T cells only, Raji only and both without anti-human CD3/anti-human CD28 were used as controls. Human PBMC derived T cells are effectively stimulated with anti-human CD3/CD28. Mean value with SD are indicated. Error bars are equivalent throughout.

Suppl Fig 4: HIS T cell stimulatory effect.

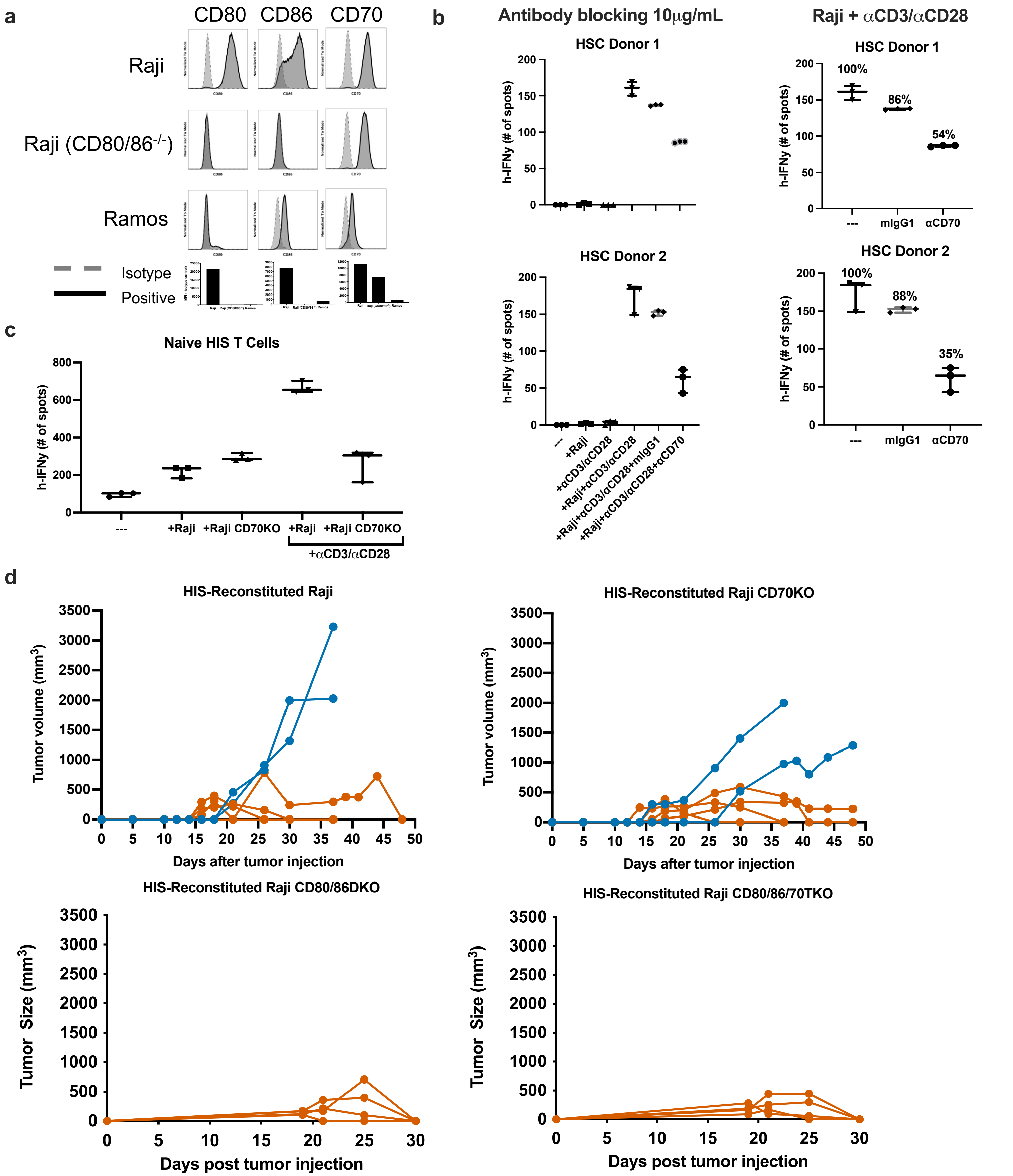

a Expression of CD80, CD86 and CD70 was visualized by flow cytometry in Raji, Raji CD80/86 DKO and Ramos. **b** Human immune system (HIS) T cells were blocked with 10mg/mL of mIgG1 or anti-CD70 in an ELISpot assay. **c** Naive HIS T cells were co-cultured along (-), Raji, Raji CD70KO, Raji with anti-CD3/anti-CD38, or Raji CD70KO with anti-CD3/anti-CD28 in an ELISpot assay. **d** HIS-reconstituted mice were were implanted subcutaneously into the right flank with Raji, Raji CD70KO, Raji CD80/86DKO or Raji CD80/86/70TKO. The number of mice with tumor progressor (P;blue) or regressor (R;orange) is shown. Mean value with SD are indicated. Error bars are equivalent throughout.

Supplementary Table 1

3 TCR sequences (flanked by XbaI and NotI sites):

clonotype	TCR sequences (flanked by XbaI and NotI sites)
TCR1	TCTAGAGCCACCATGGAAACTCTCCTGGGAGTGTCTTTGGTGATTCTATGGCTTCAACTGGCTAGGGTGAACAGTCAACAGGGA<br>GAAGAGGATCCTCAGGCCTTGAGCATCCAGGAGGGTGAAAATGCCACCATGAACTGCAGTTACAAAACTAGTATAAACAATTTA<br>CAGTGGTATAGACAAAATTCAGGTAGAGGCCTTGTCCACCTAATTTTAATACGTTCAAATGAAAGAGAGAAACACAGTGGAAGA<br>TTAAGAGTCACGCTTGACACTTCCAAGAAAAGCAGTTCCTTGTTGATCACGGCTTCCCGGGCAGCAGACACTGCTTCTTACTTCTG<br>TGCTACGATCGGCACTGCCAGTAAACTCACCTTTGGGACTGGAACAAGACTTCAGGTCACGCTCGATATCCAGAACCCTGACCCT<br>GCCGTGTACCAGCTGAGAGACTCTAAATCCAGTGACAAGTCTGTCTGCCTATTCACCGATTTTGATTCTCAAACAAATGTGTCACA<br>AAGTAAGGATTCTGATGTGTATATCACAGACAAAACTGTGCTAGACATGAGGTCTATGGACTTCAAGAGCAACAGTGCTGTGGC<br>CTGGAGCAACAAATCTGACTTTGCATGTGCAAACGCCTTCAACAACAGCATTATTCCAGAAGACACCTTCTTCCCCAGCCCAGAA<br>AGTTCCTGTGATGTCAAGCTGGTCGAGAAAAGCTTTGAAACAGATACGAACCTAAACTTTCAAAACCTGTCAGTGATTGGGTTCC<br>GAATCCTCCTCCTGAAAGTGGCCGGGTTTAATCTGCTCATGACGCTGCGGCTGTGGTCCAGCCGTGCCAAGCGATCCGGATCCG<br>GAGCCCCTGTAAAGCAGACTTTGAATTTTGACCTTCTCAAGTTGGCGGGAGACGTCGAGTCCAACCCTGGGCCCATGGGCTCCT<br>GGACCCTCTGCTGTGTGTCCCTTTGCATCCTGGTAGCAAAGCACACAGATGCTGGAGTTATCCAGTCACCCCGGCACGAGGTGAC<br>AGAGATGGGACAAGAAGTGACTCTGAGATGTAAACCAATTTCAGGACATGACTACCTTTTCTGGTACAGACAGACCATGATGCG<br>GGGACTGGAGTTGCTCATTTACTTTAACAACAACGTTCCGATAGATGATTCAGGGATGCCCGAGGATCGATTCTCAGCTAAGATG<br>CCTAATGCATCATTCTCCACTCTGAAGATCCAGCCCTCAGAACCCAGGGACTCAGCTGTGTACTTCTGTGCCAGCAGCCGTAATG<br>GGAGAAAAAACATTCAGTACTTCGGCGCCGGGACCCGGCTCTCAGTGCTGGACCTGAACAAGGTGTTCCCACCCGAGGTCGCTG<br>TGTTTGAGCCATCAGAAGCAGAGATCTCCCACACCCAAAAGGCCACACTGGTGTGCCTGGCCACAGGCTTCTTCCCTGACCACGT<br>GGAGCTGAGCTGGTGGGTGAATGGGAAGGAGGTGCACAGTGGGGTCAGCACGGACCCGCAGCCCCTCAAGGAGCAGCCCGCC<br>CTCAATGACTCCAGATACTGCCTGAGCAGCCGCCTGAGGGTCTCGGCCACCTTCTGGCAGAACCCCCGCAACCACTTCCGCTGTC<br>AAGTCCAGTTCTACGGGCTCTCGGAGAATGACGAGTGGACCCAGGATAGGGCCAAACCCGTCACCCAGATCGTCAGCGCCGAG<br>GCCTGGGGTAGAGCAGACTGTGGCTTTACCTCGGTGTCCTACCAGCAAGGGGTCCTGTCTGCCACCATCCTCTATGAGATCCTGC<br>TAGGGAAGGCCACCCTGTATGCTGTGCTGGTCAGCGCCCTTGTGTTGATGGCCATGGTCAAGAGAAAGGATTTCTGAGCGGCCG<br>C
TCR 2	TCTAGAGCCACCATGAAGACATTTGCTGGATTTTCGTTCCTGTTTTTGTGGCTGCAGCTGGACTGTATGAGTAGAGGAGAGGATG<br>TGGAGCAGAGTCTTTTCCTGAGTGTCCGAGAGGGAGACAGCTCCGTTATAAACTGCACTTACACAGACAGCTCCTCCACCTACTT<br>ATACTGGTATAAGCAAGAACCTGGAGCAGGTCTCCAGTTGCTGACGTATATTTTTCAAATATGGACATGAAACAAGACCAAAG

	ACTCACTGTTCTATTGAATAAAAAGGATAAACATCTGTCTCTGCGCATTGCAGACACCCAGACTGGGGACTCAGCTATCTACTTCT<br>GTGCAGAGAGTACCACGGGCAGGAGAGCACTTACTTTTGGGAGTGGAACAAGACTCCAAGTGCAACCAGATATCCAGAACCCT<br>GACCCTGCCGTGTACCAGCTGAGAGACTCTAAATCCAGTGACAAGTCTGTCTGCCTATTCACCGATTTTGATTCTCAAACAAATGT<br>GTCACAAAGTAAGGATTCTGATGTGTATATCACAGACAAAACTGTGCTAGACATGAGGTCTATGGACTTCAAGAGCAACAGTGC<br>TGTGGCCTGGAGCAACAAATCTGACTTTGCATGTGCAAACGCCTTCAACAACAGCATTATTCCAGAAGACACCTTCTTCCCCAGC<br>CCAGAAAGTTCCTGTGATGTCAAGCTGGTCGAGAAAAGCTTTGAAACAGATACGAACCTAAACTTTCAAAACCTGTCAGTGATTG<br>GGTTCCGAATCCTCCTCCTGAAAGTGGCCGGGTTTAATCTGCTCATGACGCTGCGGCTGTGGTCCAGCCGTGCCAAGCGATCCG<br>GATCCGGAGCCCCTGTAAAGCAGACTTTGAATTTTGACCTTCTCAAGTTGGCGGGAGACGTCGAGTCCAACCCTGGGCCCATGG<br>GGAGGTCTCAGAATGACGCCCTTGAAAGACGTGTTCCCTTTTCACCAATGCACAGACCCAGAGGACCCCTCCATCCTGCAGTTCC<br>TGCCATGAGCCTCGGGCTCCTGTGCTGTGGGGCCTTTTCTCTCCTGTGGGCAGGTCCAGTGAATGCTGGTGTCACTCAGACCCCA<br>AAATTCCGGGTCCTGAAGACAGGACAGAGCATGACACTGCTGTGTGCCCAGGATATGAACCATGAATACATGTACTGGTATCGA<br>CAAGACCCAGGCATGGGGCTGAGGCTGATTCATTACTCAGTTGGTGAGGGTACAACTGCCAAAGGAGAGGTCCCTGATGGCTA<br>CAATGTCTCCAGATTAAAAAAACAGAATTTCCTGCTGGGGTTGGAGTCGGCTGCTCCCTCCCAAACATCTGTGTACTTCTGTGCCA<br>GCAGTTGGACAGGGGGCGATGGCTACACCTTCGGTTCGGGGACCAGGTTAACCGTTGTAGACCTGAACAAGGTGTTCCCACCC<br>GAGGTCGCTGTGTTTGAGCCATCAGAAGCAGAGATCTCCCACACCCAAAAGGCCACACTGGTGTGCCTGGCCACAGGCTTCTTC<br>CCTGACCACGTGGAGCTGAGCTGGTGGGTGAATGGGAAGGAGGTGCACAGTGGGGTCAGCACGGACCCGCAGCCCCTCAAGG<br>AGCAGCCCGCCCTCAATGACTCCAGATACTGCCTGAGCAGCCGCCTGAGGGTCTCGGCCACCTTCTGGCAGAACCCCCGCAACC<br>ACTTCCGCTGTCAAGTCCAGTTCTACGGGCTCTCGGAGAATGACGAGTGGACCCAGGATAGGGCCAAACCCGTCACCCAGATCG<br>TCAGCGCCGAGGCCTGGGGTAGAGCAGACTGTGGCTTTACCTCGGTGTCCTACCAGCAAGGGGTCCTGTCTGCCACCATCCTCT<br>ATGAGATCCTGCTAGGGAAGGCCACCCTGTATGCTGTGCTGGTCAGCGCCCTTGTGTTGATGGCCATGGTCAAGAGAAAGGATT<br>TCTGAGCGGCCGC
TCR 3	TCTAGAGCCACCATGGCCTCTGCACCCATCTCGATGCTTGCGATGCTCTTCACATTGAGTGGGCTGAGAGCTCAGTCAGTGGCTC<br>AGCCGGAAGATCAGGTCAACGTTGCTGAAGGGAATCCTCTGACTGTGAAATGCACCTATTCAGTCTCTGGAAACCCTTATCTTTT<br>TTGGTATGTTCAATACCCCAACCGAGGCCTCCAGTTCCTTCTGAAATACATCACAGGGGATAACCTGGTTAAAGGCAGCTATGGC<br>TTTGAAGCTGAATTTAACAAGAGCCAAACCTCCTTCCACCTGAAGAAACCATCTGCCCTTGTGAGCGACTCCGCTTTGTACTTCTG<br>TGCTGTGAGAGCACCCCTATACAACTTCAACAAATTTTACTTTGGATCTGGGACCAAACTCAATGTAAAACCAGATATCCAGAAC<br>CCTGACCCTGCCGTGTACCAGCTGAGAGACTCTAAATCCAGTGACAAGTCTGTCTGCCTATTCACCGATTTTGATTCTCAAACAAA<br>TGTGTCACAAAGTAAGGATTCTGATGTGTATATCACAGACAAAACTGTGCTAGACATGAGGTCTATGGACTTCAAGAGCAACAG<br>TGCTGTGGCCTGGAGCAACAAATCTGACTTTGCATGTGCAAACGCCTTCAACAACAGCATTATTCCAGAAGACACCTTCTTCCCCA<br>GCCCAGAAAGTTCCTGTGATGTCAAGCTGGTCGAGAAAAGCTTTGAAACAGATACGAACCTAAACTTTCAAAACCTGTCAGTGA<br>TTGGGTTCCGAATCCTCCTCCTGAAAGTGGCCGGGTTTAATCTGCTCATGACGCTGCGGCTGTGGTCCAGCCGTGCCAAGCGATC

	CGGATCCGGAGCCCCTGTAAAGCAGACTTTGAATTTTGACCTTCTCAAGTTGGCGGGAGACGTCGAGTCCAACCCTGGGCCCAT<br>GAGGTCTCAGAATGACTTCCTTGAGAGTCCTGCTCCCCTTTCATCAATGCACAGATACAGAAGACCCCTCCGTCATGCAGCATCT<br>GCCATGAGCATCGGCCTCCTGTGCTGTGCAGCCTTGTCTCTCCTGTGGGCAGGTCCAGTGAATGCTGGTGTCACTCAGACCCCAA<br>AATTCCAGGTCCTGAAGACAGGACAGAGCATGACACTGCAGTGTGCCCAGGATATGAACCATGAATACATGTCCTGGTATCGAC<br>AAGACCCAGGCATGGGGCTGAGGCTGATTCATTACTCAGTTGGTGCTGGTATCACTGACCAAGGAGAAGTCCCCAATGGCTACA<br>ATGTCTCCAGATCAACCACAGAGGATTTCCCGCTCAGGCTGCTGTCGGCTGCTCCCTCCCAGACATCTGTGTACTTCTGTGCCAGC<br>AGTTACGGCAGGGGGCGTGAGCAGTACTTCGGGCCGGGCACCAGGCTCACGGTCACAGACCTGAACAAGGTGTTCCCACCCGA<br>GGTCGCTGTGTTTGAGCCATCAGAAGCAGAGATCTCCCACACCCAAAAGGCCACACTGGTGTGCCTGGCCACAGGCTTCTTCCCT<br>GACCACGTGGAGCTGAGCTGGTGGGTGAATGGGAAGGAGGTGCACAGTGGGGTCAGCACGGACCCGCAGCCCCTCAAGGAGC<br>AGCCCGCCCTCAATGACTCCAGATACTGCCTGAGCAGCCGCCTGAGGGTCTCGGCCACCTTCTGGCAGAACCCCCGCAACCACTT<br>CCGCTGTCAAGTCCAGTTCTACGGGCTCTCGGAGAATGACGAGTGGACCCAGGATAGGGCCAAACCCGTCACCCAGATCGTCAG<br>CGCCGAGGCCTGGGGTAGAGCAGACTGTGGCTTTACCTCGGTGTCCTACCAGCAAGGGGTCCTGTCTGCCACCATCCTCTATGA<br>GATCCTGCTAGGGAAGGCCACCCTGTATGCTGTGCTGGTCAGCGCCCTTGTGTTGATGGCCATGGTCAAGAGAAAGGATTTCTG<br>AGCGGCCGC
