# Supplementary material for: Spontaneous tumor regression mediated by human T cells in a humanized immune system mouse model
Source: Commun Biol. 2023 Apr 22;6:444. doi: 10.1038/s42003-023-04824-z (PMC10122651; doi:10.1038/s42003-023-04824-z)
Supplement: Supplementary file 2 — Description of Additional Supplementary Files [file 42003_2023_4824_MOESM2_ESM.pdf]

Description of Additional Supplementary Files

File name: Supplementary Data

Description: Source data for figures
