# Supplementary material for: Spontaneous tumor regression mediated by human T cells in a humanized immune system mouse model
Source: Commun Biol. 2023 Apr 22;6:444. doi: 10.1038/s42003-023-04824-z (PMC10122651; doi:10.1038/s42003-023-04824-z)
Supplement: Supplementary file 4 — Reporting Summary [file 42003_2023_4824_MOESM4_ESM.pdf]

nature portfolio

Corresponding author(s): Davor Frleta

Last updated by author(s): Nov 14, 2022

Reporting Summary

Nature Portfolio wishes to improve the reproducibility of the work that we publish. This form provides structure for consistency and transparency in reporting. For further information on Nature Portfolio policies, see our Editorial Policies and the Editorial Policy Checklist.

Statistics

For all statistical analyses, confirm that the following items are present in the figure legend, table legend, main text, or Methods section.

n/a	Confirmed	
☐	☒	The exact sample size (*n*) for each experimental group/condition, given as a discrete number and unit of measurement
☐	☒	A statement on whether measurements were taken from distinct samples or whether the same sample was measured repeatedly
☐	☒	The statistical test(s) used AND whether they are one- or two-sided<br>*Only common tests should be described solely by name; describe more complex techniques in the Methods section.*
☒	☐	A description of all covariates tested
☒	☐	A description of any assumptions or corrections, such as tests of normality and adjustment for multiple comparisons
☐	☒	A full description of the statistical parameters including central tendency (e.g. means) or other basic estimates (e.g. regression coefficient) AND variation (e.g. standard deviation) or associated estimates of uncertainty (e.g. confidence intervals)
☒	☐	For null hypothesis testing, the test statistic (e.g. *F*, *t*, *r*) with confidence intervals, effect sizes, degrees of freedom and *P* value noted<br>*Give P values as exact values whenever suitable.*
☒	☐	For Bayesian analysis, information on the choice of priors and Markov chain Monte Carlo settings
☒	☐	For hierarchical and complex designs, identification of the appropriate level for tests and full reporting of outcomes
☒	☐	Estimates of effect sizes (e.g. Cohen's *d*, Pearson's *r*), indicating how they were calculated

Our web collection on statistics for biologists contains articles on many of the points above.

Software and code

Policy information about availability of computer code

Data collection	No software was used
Data analysis	No software was used

For manuscripts utilizing custom algorithms or software that are central to the research but not yet described in published literature, software must be made available to editors and reviewers. We strongly encourage code deposition in a community repository (e.g. GitHub). See the Nature Portfolio guidelines for submitting code & software for further information.

Data

Policy information about availability of data

All manuscripts must include a data availability statement. This statement should provide the following information, where applicable:

- Accession codes, unique identifiers, or web links for publicly available datasets
- A description of any restrictions on data availability
- For clinical datasets or third party data, please ensure that the statement adheres to our policy

Data availability statement

All data generated or analyzed during this study are included in this published article (and its supplementary information files). Please reach out to corresponding

author for any material requests.

Human research participants

Policy information about studies involving human research participants and Sex and Gender in Research.

Reporting on sex and gender	No gender information was collected in use of human HSC donors.
Population characteristics	See above
Recruitment	Human tissue sample was purchased through Advanced Biosciences Resources (Alameda, CA) which obtained tissue with proper consent and compliance.
Ethics oversight	Compliance and consent was seen by Advanced Biosciences Resources

Note that full information on the approval of the study protocol must also be provided in the manuscript.

Field-specific reporting

Please select the one below that is the best fit for your research. If you are not sure, read the appropriate sections before making your selection.

☒ Life sciences ☐ Behavioural & social sciences ☐ Ecological, evolutionary & environmental sciences

For a reference copy of the document with all sections, see nature.com/documents/nr-reporting-summary-flat.pdf

Life sciences study design

All studies must disclose on these points even when the disclosure is negative.

Sample size	No sample size calculation was performed. Samples per experiment were based on availability of engrafted HIS mice and experiments were repeated at least 3 times (with different human HSC donors for engraftment) to show reproducibility.
Data exclusions	No data was excluded from analysis
Replication	Experimental findings were replicated by repeating experiment at least 3 times with HIS mice engrafted with different human HSC donors.
Randomization	Samples were randomized in regards to hCD45+ engraftment level, i.e. mice used per experiment were randomized based on level of hCD45+ cells in the blood.
Blinding	In vivo studies were blinded: Tumor measurement was done without knowledge of hCD45+ levels or T cells in mice as well as without knowledge of treatment groups e.g. specific T cell depletion. In vitro studies: single cell molecular analysis of T cells was blinded because performed by group without information on specific samples (tumor regressors, controls, etc.). In contrast ELISpot and flow cytometry could not be blinded because analysis was meant to be comparative between tumor regression and controls so individual performing the experiment knew what type of sample they were processing for analysis.

Reporting for specific materials, systems and methods

We require information from authors about some types of materials, experimental systems and methods used in many studies. Here, indicate whether each material, system or method listed is relevant to your study. If you are not sure if a list item applies to your research, read the appropriate section before selecting a response.

Materials & experimental systems

n/a	Involved in the study	
☐	☒	Antibodies
☐	☒	Eukaryotic cell lines
☒	☐	Palaeontology and archaeology
☐	☒	Animals and other organisms
☒	☐	Clinical data
☒	☐	Dual use research of concern

Methods

n/a	Involved in the study	
☒	☐	ChIP-seq
☐	☒	Flow cytometry
☒	☐	MRI-based neuroimaging

Antibodies

Antibodies used	For engraftment analysis: anti-mouse CD45-APC-Cy7 (clone 30-F11; BD Biosciences), anti-human PE-Cy5.5(clone HI30; Invitrogen), anti-human CD3-Pacific Blue (clone S4.1; Invitrogen), anti-human NKp46-APC (clone 9E2; BD Biosciences), anti-human CD14-PE-Cy7 (clone M5E2; BD Biosciences), anti-human PD-1-BV605 (clone EH12; BD Biosciences), Depletion antibodies: anti-human CD4 (depleting; clone OKT4; BioXCell), anti-human CD8 (depleting; clone OKT8; BioXCell), IFNg ELISpot: anti-human IFNg (Human IFNg ELISpot pair; BD Biosciences Cat # 551873)
Validation	Antibodies for engraftment have previously been validated. Thousands of HIS mice have been generated at Regeneron and engraftment levels checked by the antibodies described. anti-human CD4/CD8 depleting antibodies and antibodies used for IFNg ELISpot have previously been validated by manufacturer.

Eukaryotic cell lines

Policy information about cell lines and Sex and Gender in Research

Cell line source(s)	Raji (human Burkitt's lymphoma line, established from human male)<br>Ramos (human Burkitt's lymphoma line, established from human male)
Authentication	Both Raji and Ramos have previously been showed to form tumors in HIS mice, both by investigators at Regeneron as well as external investigators.
Mycoplasma contamination	Not tested for mycoplasma contamination.
Commonly misidentified lines<br>(See ICLAC register)	No commonly misidentified cell lines used.

Animals and other research organisms

Policy information about studies involving animals; ARRIVE guidelines recommended for reporting animal research, and Sex and Gender in Research

Laboratory animals	SRG: Balb/cAnN;129S4/SvJae - Rag2-/- human Sirpa IL-2Rg-/-<br>SRG-15: Balb/cAnN;129S4/SvJae - Rag2-/-, human Sirpa, IL-2Rg-/-, human IL-15
Wild animals	No wild animals used
Reporting on sex	Information on sex of animals was not collected. Both male and female animals were used with no obvious differences noted.
Field-collected samples	Study did not involve field-collected samples
Ethics oversight	Regeneron Institutional Animal Care and Use Committee

Note that full information on the approval of the study protocol must also be provided in the manuscript.

Flow Cytometry

Plots

Confirm that:

☐ The axis labels state the marker and fluorochrome used (e.g. CD4-FITC).

☒ The axis scales are clearly visible. Include numbers along axes only for bottom left plot of group (a 'group' is an analysis of identical markers).

☒ All plots are contour plots with outliers or pseudocolor plots.

☒ A numerical value for number of cells or percentage (with statistics) is provided.

Methodology

Sample preparation	Cell suspension was prepared by homogenization of tumor and spleen tissue from HIS mice. When whole blood was collected, red blood cells were lysed with ACK lysing buffer (Gibcoi). All cells were treated and FACS stained in PBS +2mM EDTA+2% fetal bovine serum. Cells were washed 3x and then fixed in 2% paraformaldehyde before being analyzed.
Instrument	Becton-Dickinson FACSymphony A3 Cell Analyzer
Software	FlowJo Version 10.8
Cell population abundance	Collected at least 50,000 T cells for analysis

Gating strategy

Engraftment analysis: human CD45+/mouse CD45- singlets analyzed for percentage of human CD3+ (T cells), human CD19+ (B cells), human NKp46+ (NK cells), and human CD14+ (myeloid cells). For T cell analysis: human CD45+/mouse CD45-/ human CD3+ cells when analyzing T cells. Further segregated that population into human CD4+ or human CD8+ gates to analyze MFI of PD-1 and HLA-DR expression.

☐ Tick this box to confirm that a figure exemplifying the gating strategy is provided in the Supplementary Information.
